# Effects of whole-brain radiation therapy on the blood–brain barrier in immunocompetent and immunocompromised mouse models

**DOI:** 10.1186/s13014-023-02215-6

**Published:** 2023-02-03

**Authors:** K. E. Blethen, S. A. Sprowls, T. A. Arsiwala, C. P. Wolford, D. M. Panchal, R. A. Fladeland, M. J. Glass, L. P. Dykstra, B. N. Kielkowski, J. R. Blackburn, C. J. Andrick, P. R. Lockman

**Affiliations:** 1 Department of Pharmaceutical Sciences, School of Pharmacy, West Virginia University, 108 Biomedical Drive, Morgantown, WV 26506 USA; 2 Department of Cardiovascular and Metabolic Sciences, Lerner Research Institute, Cleveland Clinic, Cleveland, OH USA; 3 Department of Chemical and Biomedical Engineering, Benjamin M. Statler College of Engineering and Mineral Resources, West Virginia University, Morgantown, WV USA; 4 Laboratory of Cancer Biology and Genetics, Center for Cancer Research, National Cancer Institute, Bethesda, MD USA

**Keywords:** Neuroinflammation, Immunotherapy, Brain metastases, Glioblastoma, Immune response, Vascular permeability, Whole brain irradiation, Radiotherapy, Efflux transporter

## Abstract

**Background:**

Approximately 20% of all cancer patients will develop brain metastases in their lifespan. The standard of care for patients with multiple brain metastases is whole-brain radiation therapy, which disrupts the blood–brain barrier. Previous studies have shown inflammatory mediators play a role in the radiation-mediated increase in permeability. Our goal was to determine if differential permeability post-radiation occurs between immunocompetent and immunocompromised mice.

**Methods:**

We utilized a commissioned preclinical irradiator to irradiate brains of C57Bl/6J wild-type and athymic nude mice. Acute (3–24 h) effects on blood–brain barrier integrity were evaluated with our *in-situ* brain perfusion technique and quantitative fluorescent and phosphorescent microscopy. The presence of inflammatory mediators in the brain and serum was determined with a proinflammatory cytokine panel.

**Results:**

Blood–brain barrier integrity and efflux transporter activity were altered in the immunocompetent mice 12 h following irradiation without similar observations in the immunocompromised mice. We observed increased TNF-α concentrations in the serum of wild-type mice immediately post-radiation and nude mice 12 h post-radiation. The brain concentration of CXCL1 was also increased in both mouse strains at the 12-h time point.

**Conclusions:**

The immune response plays a role in the magnitude of blood–brain barrier disruption following irradiation in a time- and size-dependent manner.

**Supplementary Information:**

The online version contains supplementary material available at 10.1186/s13014-023-02215-6.

## Background

The blood–brain barrier (BBB) is a selectively permeable, tightly regulated physiochemical barrier between the blood and brain parenchyma [1]. Endothelial cells are locked together by tight junction proteins and sheathed by a basement membrane embedded with pericytes. Astrocyte foot processes encompass the outermost layer. These components work together to regulate molecule passage into the brain [2]. Lipophilic molecules may diffuse across the barrier, but efflux transporter pumps, including P-glycoprotein (P-gp) and breast cancer resistance protein (BCRP), actively shuttle molecules back into the systemic blood circulation [3]. During metastasis, cancer cells infiltrate the brain parenchyma and as they grow displace BBB components resulting in a “leaky” blood-tumor barrier (BTB). Although the BTB is more permeable than BBB, it does not allow drug accumulation in cytotoxic concentrations at the tumor bed [4, 5]. This is one reason for poor prognosis and treatment failure among patients with brain metastases [6].

Treatment for brain metastases includes a combination of radiation therapy, chemotherapy, and/or surgical resection. Depending on the size and number of metastases, patients receive stereotactic radiosurgery (SRS) and/or whole-brain radiation therapy (WBRT) [7]. In the case of multiple metastases, WBRT is most commonly administered as 30 Gy in 10 fractions. While this improves overall survival, cognitive decline is often seen within six months of treatment.

The timing and magnitude of WBRT vascular permeability changes are not well defined. Unfortunately, most studies have used variable radiotherapy doses and timeframes, leading to results which cannot be easily compared [8–13]. Nevertheless, for the literature that is consistent it appears that BBB disruption occurs anywhere between 24 h and 4 weeks following radiotherapy [14–16].

Multiple studies have shown a synergistic effect of combining radiotherapy with immunotherapy [17–22]. A recent report evaluating immunotherapy efficacy after SRS demonstrated patients with melanoma brain metastases had better outcomes if the immunotherapy was delivered within 7 days of radiation [23]. Optimal timing of immunotherapy administration with radiation is unclear and varies between tumor type, but is important to elicit a robust immune response [24]. Furthermore, local radiotherapy to the primary tumor can benefit brain metastases via an abscopal effect, which is hypothesized to be associated with heightened anti-tumor immunity [25]. Whole-brain radiation therapy induces neuroinflammation with aggregation of immune cells along the BBB and increased proinflammatory mediators in the brain, such as TNF-α. Blocking TNF-α with a recombinant antibody reduces BBB permeability changes following WBRT in mice [10]. This provides evidence of the inflammatory response to radiation playing a role in the magnitude of BBB disruption. Majority of brain metastasis animal models are immunocompromised [26] and this may affect studies investigating BBB/BTB permeability following WBRT.

This study aims to determine the effect of the immune response in radiation-mediated BBB disruption. Herein we evaluated BBB disruption and immune responses in immunocompetent and athymic nude immunocompromised mice with one dose of 15.5 Gy, which has a biological effective dose (BED) of 39.5 Gy. This is similar to the BED of the clinical treatment regimen (30 Gy in 10 fraction, BED = 39) [27, 28]. We hypothesized that WBRT in immunocompetent mice would result in a higher magnitude of BBB permeability in comparison to athymic immunocompromised mice. We utilized in situ brain perfusions and quantitative fluorescent and phosphorescent imaging to identify BBB permeability changes within 24 h following WBRT. A time- and size-dependent opening of the BBB following WBRT in immunocompetent mice was observed, without similar observations in immunocompromised mice. We evaluated presence of proinflammatory mediators in the brain and serum of immunocompetent and immunocompromised mice post-WBRT and observed increased TNF-α serum concentrations and CXCL1 brain concentrations in both strains. These data suggest the immune response may play a role in the magnitude and timing of BBB disruption following WBRT.

## Methods

### Animals

All animal experiments were approved by the Institutional Animal Care and Use Committee at West Virginia University. Female C57Bl/6 and C57Bl/6 athymic nude mice were purchased from Jackson Laboratory (Bar Harbor, ME). All animals were approximately 8–10 weeks of age and 25 g during experiments. Animals were allowed to acclimate for at least one week prior to experimentation.

### Irradiation protocol

The XenX irradiator (Xstrahl, Suwanee, GA) at West Virginia University was commissioned to deliver accurate, clinically-relevant doses of radiation as previously described [29]. C57Bl/6 and C57Bl/6 athymic nude mice were anesthetized with 1–3% isoflurane. All animals were treated with whole-brain irradiation except animals administered 3 kDa Texas Red dextran and ^14^C-AIB tracers, which were treated with irradiation only on the right hemisphere. Sham control mice were anesthetized with 1–3% isoflurane and placed into the XenX irradiator for the same amount of time it takes to dose mice with 15.5 Gy (~ 5.5 min).

### In-situ brain perfusion technique

The *in-situ* brain perfusion technique was modified from Takasato et al. [30, 31]. Physiological buffer (2.4 nM NaH_2_PO_4_, 4.2 mM KCl, 24 mM NaHCO_3_, 128 mM NaCl, 1.5 mM CaCl_2_, 0.9 mM MgCl_2_ and 9 mM D-glucose) with ^14^C-sucrose (Moravek Biochemicals, Brea, CA) and ^3^H-ivermectin (Moravek Biochemicals, Brea, CA) was prepared, filtered, and heated to 37 °C. At various time points (3–24 h), mice were anesthetized with ketamine (75–100 mg/kg) and xylazine (6–8 mg/kg) followed by whole brain perfusion for two minutes. Brains were collected and sectioned into cortical tissue, subcortical tissue, cerebellum, and brain stem. Sections were weighed and digested with 3 mL Solvable (PerkinElmer, Waltham, MA) in scintillation vials overnight at 55 °C. UltimaGold LSC Cocktail (PerkinElmer, Waltham, MA) was added to samples, vortexed, and read on a Tri-Carb Liquid Scintillation Counter (PerkinElmer, Waltham, MA). Integrity of the BBB is reported as increases in the vascular space (mL/g) while ivermectin uptake/efflux transporter activity is reported as unidirectional transfer constant, K_in_ (mL/s/g). The vascular volume and K_in_ were calculated as described previously with the equation below [30].

$$\frac{{Q}^{*}}{{C}^{*}}={K}_{in}\left(T\right)+{V}_{0}$$

### Tracer administration and brain processing

TxRd 3 kDa dextran (Invitrogen, Waltham, MA) and ^14^C-α-aminoisobutyric acid (AIB) (American Radiolabeled Chemicals, Saint Louis, MO) were injected in concentrations of 6 mg/kg and 100 µCi/animal respectively via tail vein and circulated for 10 min. Brains were collected and snap-frozen in isopentane then stored at −20 °C until sliced. Frozen brains were sliced at 20 µm thickness using a Leica CM3050 cryostat (Leica Microsystems, Los Angeles, CA).

### Fluorescent imaging, phosphorescent imaging, and analysis

Fluorescent analyses were performed using an Olympus MVX10 stereomicroscope (Olympus, Tokyo, Japan) (optical zoom range 0.63–12.6, NA = 0.5) with a Hamamatsu ORCA Flash4.0 v2 sCMOS and DAPI/FITC/RFP/Cy5/Cy7 filter set. Sections were imaged using RFP (588 nm) channel to detect 3 kDa Texas red (TxRd) dextran. CellSens image analysis software (Olympus, Tokyo, Japan) was used to quantitate 3 kDa TxRd dextran accumulation. The same slides were placed in quantitative autoradiography cassettes (GE Healthcare Life Sciences, Chicago, IL) with corresponding ^14^C standards (0.1–862 nCi/g). A 20 × 40 super-resolution phosphor screen (Fujifilm Life Sciences, Cambridge, MA) was placed over the slides and developed for 21 days. Screens were read on FUJI FLA-7000 (Fujifilm Life Sciences, Cambridge, MA) high-resolution phosphor imager. Quantification of ^14^C-AIB was analyzed with MCID Analysis Software (InterFocus Imaging, Cambridge, England). Accumulation of ^14^C-AIB is reported as nCi/g while 3 kDa TxRd accumulation is sum intensity/area.

### Cytokine protein quantification

Sample preparation for V-PLEX Proinflammatory Panel 1 Mouse kit (Meso Scale Diagnostics, Rockville, MD) was described previously [32]. Brains were collected and snap-frozen in isopentane then stored at −20 °C until time of homogenization. Brains were homogenized in RIPA buffer (Thermo Fisher Scientific, Waltham, MA) with Halt Protease and Phosphatase Inhibitor Cocktail (Thermo Fisher Scientific, Waltham, MA). Samples were centrifuged at 13,300 RPM for 15 min at 4 °C. Supernatant was collected and stored at −20 °C. Pierce BCA Protein Assay was performed to determine total protein concentration. Samples were diluted and loaded on the MSD plate at the same total protein concentrations. Manufacturer’s protocol for the V-PLEX Proinflammatory Panel 1 Mouse kit was followed as described. Plates were read with Meso Quickplex SQ 120 (Meso Scale Diagnostics, Rockville, MD) and data was analyzed via MSD Discovery Workbench software (Meso Scale Diagnostics, Rockville, MD). Concentrations of cytokines are presented as pg/mL.

### Statistical analysis

Data were analyzed and plotted with GraphPad Prism 8 software (GraphPad Software, San Diego, CA). Results are presented as mean ± SEM unless noted otherwise. Statistical differences between two groups were assessed using Student’s t test. One-way ANOVA with a Tukey posttest was utilized for data with more than two groups. Differences were considered statistically significant at *p* < 0.05 (*).

## Results

### BBB disruption and dysfunctional efflux transporter activity following WBRT in immunocompetent mice

**Fig. 1:**
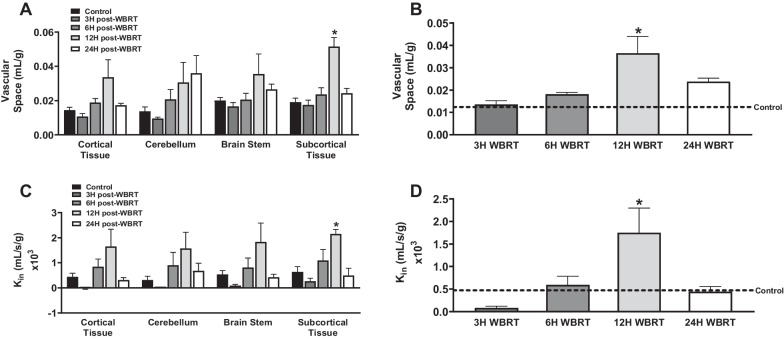
Disruption of BBB homeostasis following WBRT in immunocompetent mice. **A**–**D** Significant increase in ^14^C-sucrose (**A**, **B**) and ^3^H-ivermectin (**C**, **D**) uptake observed regionally and in whole brains 12 h following WBRT (15.5 Gy) in wild-type C57Bl/6 mice (*p* < 0.05)

**Fig. 2:**
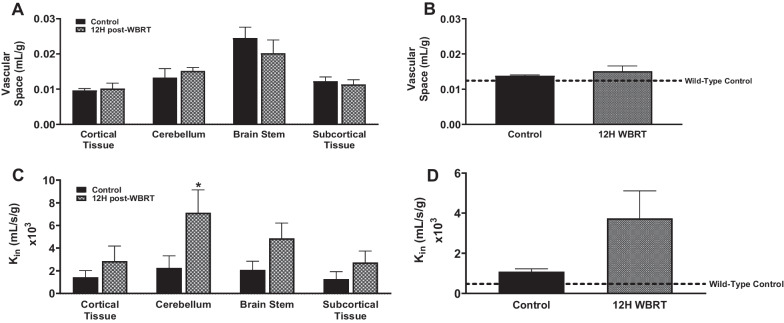
No significant changes in BBB homeostasis following WBRT in whole brains of immunocompromised mice. **A**–**D** No significant differences in ^14^C-sucrose uptake **A**–**B** regionally or in the whole brains of nude mice 12 h following WBRT. Significant increase in.^3^H-ivermectin **C**, **D** uptake in the cerebellum of nude mice 12 h post-WBRT, but no significant changes in whole brain uptake (*p* < 0.05)

To evaluate BBB permeability and efflux transporter function after WBRT, we performed *in-situ* brain perfusions with trace amounts of radiolabeled ^14^C-sucrose, an impermeable marker of BBB integrity, and ^3^H-ivermectin, an efflux transporter substrate, in physiological buffer at various time points (3–24 h) post-radiation. We observed a significant increase in ^14^C-sucrose and ^3^H-ivermectin brain uptake 12 h post-WBRT in immunocompetent C57Bl/6 mice (Fig. 1). The ^14^C-sucrose uptake of wild-type (WT) control mice was 1.4 ± 0.2 × 10^–5^ mL/g and 12 h post-WBRT it was significantly higher (*p* < 0.05) at 3.6 ± 0.8 × 10^–5^ mL/g. In a similar manner, the uptake of ^3^H-ivermectin in WT control mice (4.7 ± 1.5 × 10–4 mL/s/g) significantly increased (*p* < 0.05) to 1.7 ± 0.6 × 10^–3^ mL/s/g 12 h after being treated with WBRT. No significant differences in ^14^C-sucrose or ^3^H-ivermectin whole brain uptake were observed in athymic nude C57Bl/6 mice (Fig. 2). Athymic nude control mice had a ^14^C-sucrose uptake of 1.4 × 10^–5^ mL/g and 12 h post-WBRT ^14^C-sucrose uptake (1.5 ± 0.2 × 10^–5^ mL/g) remained non-significantly altered. The uptake of ^3^H-ivermectin did not significantly change between the athymic nude control and 12 h post-WBRT mice, 1.1 ± 0.1 × 10^–3^ mL/s/g and 3.7 ± 1.4 × 10^–3^ mL/s/g, respectively. We did not observe significant differences in the two mouse strains baseline ^14^C-sucrose uptake, however, there was a significant increase (*p* < 0.05) in the uptake of ^3^H-ivermectin in the athymic nude C57Bl/6 mice (Additional file 1: Fig. S1).

### Time- and size-dependent opening of the BBB post-WBRT

**Fig. 3:**
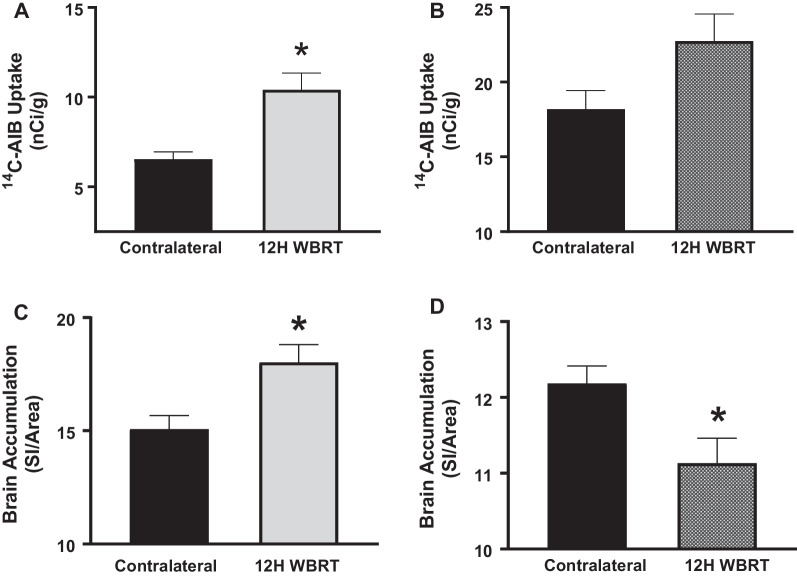
Increased BBB permeability following WBRT in immunocompetent mice is time and size-dependent. **A**–**D** Significant increase in ^14^C-AIB (**A**) and 3 kDa TxRd (**C**) uptake observed 12 h following WBRT (15.5 Gy) in wild-type C57Bl/6 mice, but not in athymic nude C57Bl/6 mice (**B**, **D**) (*p* < 0.05)

To confirm the BBB permeability alterations observed above, we completed additional experiments where we injected ^14^C-AIB (~ 103 Da) and TxRd dextran (3 kDa) at various time points (3–24 h) after half-brain irradiation (15.5 Gy). Uptake of ^14^C-AIB and TxRd dextran in the irradiated side of the brain were compared to the contralateral side. A significant increase in ^14^C-AIB and TxRd dextran uptake was noted 12 h post-radiation in the immunocompetent mice (Fig. 3A, C). Brain accumulation of ^14^C-AIB at 12 h significantly increased (*p* < 0.05) to 11.0 ± 1.3 nCi/g in the treated side compared to 7.2 ± 0.6 nCi/g in the contralateral side. For the larger tracer, TxRd 3 kDa dextran, accumulation in the contralateral side of the WT brains was 15.1 ± 0.6 SI/area while the irradiated side had a significant increase (*p* < 0.05) of 18.0 ± 0.8 SI/area. Uptake of ^14^C-AIB was fivefold higher than TxRd 3 kDa dextran in WT brains 12 h post-irradiation. No differences were observed in ^14^C-AIB brain uptake in the athymic nude mice (Fig. 3B). Accumulation of ^14^C-AIB in nude mice on the untreated side was 18.2 ± 1.2 nCi/g and did not significantly increase with radiation (22.7 ± 1.8 nCi/g). We observed a decrease in 3 kDa TxRd dextran uptake in athymic nude C57Bl/6 mice 12H post-WBRT (Fig. 3D). The contralateral side of nude mice brains was 12.2 ± 0.2 SI/area and significantly decreased (*p* < 0.05) to 11.1 ± 0.3 SI/area 12 h post-WBRT.

### Alterations in proinflammatory cytokines in the brain and serum after WBRT

**Fig. 4:**
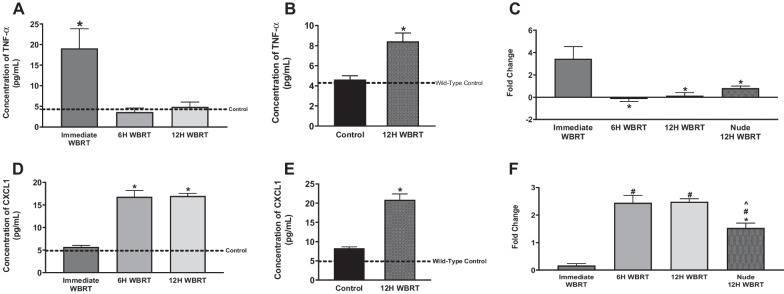
Increased proinflammatory mediators following WBRT in immunocompetent and immunocompromised mice. **A**–**F** Significant increase of TNF-α in the serum of WT mice immediately after WBRT (15.5 Gy) (**A**). Significant increase of TNF-α in the serum of athymic nude mice 12H post-WBRT (**B**). The fold changes of TNF-α concentrations in the serum following WBRT are significantly decreased 6H and 12H in WT mice and 12H in nude mice in comparison to WT immediate concentrations (**C**). CXCL1 is significantly increased in the brains of WT mice 6 and 12 h post-WBRT and in the brains of nude mice 12H post-WBRT (**D**, **E**). The fold changes of CXCL1 brain concentrations are significantly different between 12H timepoints of WT and nude strains (F) (*p* < 0.05)

**Fig. 5:**
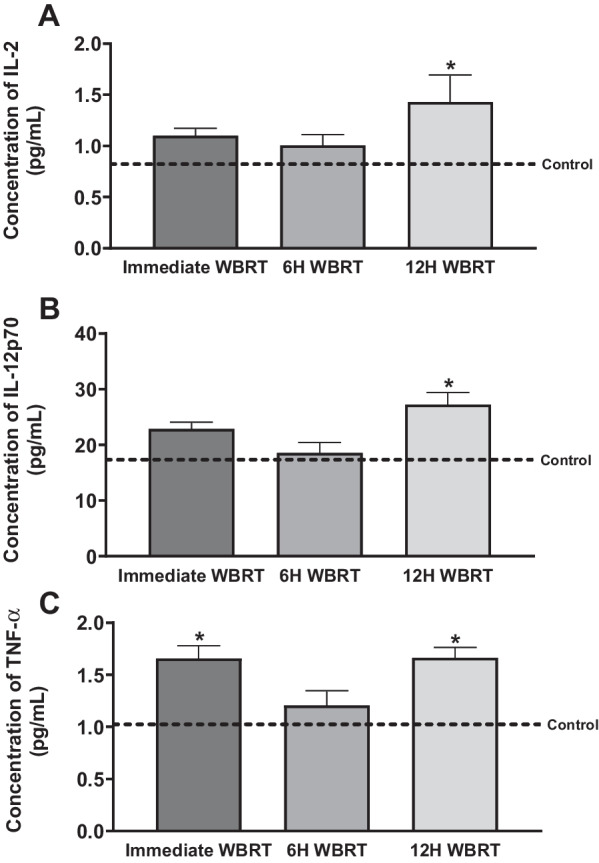
Proinflammatory cytokines significantly increased in brains of immunocompetent mice post-WBRT. **A**–**C** IL-2 and IL-12p70 are significantly increased 12H post-WBRT in brains of wild-type mice (**A**, **B**). TNF-α is significantly increased immediately and 12H post-WBRT (15.5 Gy) in brains of wild-type mice (**C**) (*p* < 0.05)

Radiation induces changes in cytokine concentrations in the brain and serum [33]. To evaluate presence of proinflammatory mediators in immunocompetent and immunocompromised mice post-WBRT, we assessed cytokine concentrations in the serum and brain with a proinflammatory MSD kit, which measures concentrations of 10 proinflammatory cytokines. The TNF-α serum concentrations were significantly increased in immunocompetent and immunocompromised mice (Fig. 4A–C). Concentration of TNF-α in the serum of WT mice immediately after WBRT was significantly higher (*p* < 0.05) than the control serum (19.0 ± 4.8 pg/mL and 4.3 ± 0.1 pg/mL, respectively) (Fig. 4A). In the nude mice, we observed an increase in TNF-α serum concentration at 12 h following radiation; control mice had 4.6 ± 0.4 pg/mL and treated mice significantly increased (p < 0.05) to 8.4 ± 0.8 pg/mL (Fig. 4B). When comparing fold changes between strains, WT mice immediately following radiation had 3.4 ± 1.1-fold higher concentration of TNF-α in serum and nude mice 12 h post-WBRT was significantly lower (*p* < 0.05) at 0.8 ± 0.2-fold (Fig. 4C). There were no significant differences in the serum concentrations of IL-1β, IL-2, IL-5, IL-6, IL-10, or CXCL1 in the WT or athymic nude mice following WBRT (Additional file 4: Fig. S4, Additional file 5: Fig. S5). The chemokine CXCL1 was significantly increased (*p* < 0.05) in the brains of WT mice 6 and 12 h post-WBRT with concentrations of 16.8 ± 1.4 pg/mL and 17.0 ± 0.5 pg/mL, respectively, in comparison to control concentration of 4.9 ± 0.4 pg/mL (Fig. 4D). The concentration of CXCL1 also increased in the athymic nude C57Bl/6 mice 12 h post-WBRT (Fig. 4E). Control nude mice had a CXCL1 brain concentration of 8.2 ± 0.4 pg/mL and increased significantly (*p* < 0.05) 12 h post-WBRT to a concentration of 20.9 ± 1.5 pg/mL. There was a significant difference (*p* < 0.05) in the fold-changes between the 12 h time points of the WT and athymic nude mice, 2.5 ± 0.1-fold and 1.5 ± 0.2-fold, respectively (Fig. 4F). Other cytokines significantly increased (*p* < 0.05) in the brains of WT mice following WBRT were TNF-α, IL-2, and IL-12p70 (Fig. 5). There were no significant differences in brain concentrations of IL-1β, IL-4, IL-5, IL-6, or IFN-γ in the WT or nude mice post-WBRT (Additional file 6: Fig. S6, Additional file 7: Fig. S7). Additionally, no differences were observed in brain concentrations of IL-2, IL-10, IL-12p70, or TNF-α in nude mice 12 h following radiation (Additional file 7: Fig. S7).

## Discussion

Whole-brain radiation therapy promotes neuroinflammation and disrupts the BBB. This is demonstrated by increased expression of proinflammatory mediators and decreased expression of tight junction proteins [34]. The majority of work in the preclinical cancer research field is performed with athymic nude mice which lack functional T-cells [35]. It is important to determine the effects WBRT may have on the BBB in athymic mice and immunocompetent mice, considering patient immune profiles lie somewhere in between during cancer treatment [36, 37]. Understanding the immune response to WBRT and the downstream effects on BBB permeability may be useful when designing treatment plans with concurrent immunotherapy, targeted therapy, or chemotherapy. Herein, we investigate the relationship between the immune response to WBRT and the effects on BBB integrity in a time- and size-dependent manner.

We observed in immunocompetent and athymic immunocompromised mice BBB integrity was intact prior to WBRT, however, we observed disruption in the WT mice 12 h post-WBRT. These findings suggest radiation-mediated BBB permeability may be impacted by the presence of functional T-cells. T-cell dependent neuroinflammation and BBB disruption have been reported in neurodegenerative diseases, ischemic stroke, and chronic stress [38–41]. Interestingly, one study observed that transfer of non-CNS-specific activated T-cells into mice results in similar levels of BBB disruption as transfer of CNS-specific activated T-cells, a model of multiple sclerosis [42].

In contrast to the BBB integrity, we observed a decrease in efflux transporter activity in the athymic nude mice at baseline. A significant decrease in efflux activity 12 h post-WBRT was observed only in the WT mice, which returned to baseline 24 h post-radiation. Numerous studies have demonstrated proinflammatory mediators alter expression and functional activity of efflux transporters in vitro and in vivo [43]. Dysfunctional efflux transport activity has also been observed in patients with Alzheimer’s disease. One study reported patients have efflux transport activity levels similar to those with pharmacological inhibitors [44, 45]. Neuroinflammation and presence of amyloid-β plaques are hallmarks of Alzheimer’s disease. It is hypothesized that the reduced efflux transporter activity contributes to poor clearance of amyloid-β from the brain, leading to plaque accumulation. Our data correspond with the current literature and indicate an immune response-dependent window of time during which efflux transporter function is decreased after WBRT.

We confirmed BBB disruption following irradiation with quantitative imaging of fluorescent and radiolabeled molecules of two different sizes. The small radiolabeled molecule, ^14^C-AIB (~ 103 Da), was fivefold higher in irradiated sides of WT mouse brains 12 h post-WBRT in comparison to the larger molecule, 3 kDa TxRd dextran. Increased accumulation of the molecules was not observed in the athymic nude mice treated with WBRT. We have previously characterized size-dependent BBB/BTB disruption in our brain tumor models and healthy mice treated with low intensity focused ultrasound [46, 47]. In both cases, molecules with lower molecular weights accumulate in the brain to a higher degree than high molecular weight molecules. These data add to our previous work and demonstrate the impact of the immune response to WBRT on BBB permeability is time- and size-dependent.

The presence of proinflammatory mediators in the serum and brain following WBRT was measured in the two mouse strains. We observed a significant increase in TNF-α in the serum of WT mice immediately following radiation and athymic nude mice 12 h post-WBRT. The increased fold change of TNF-α in WT mice immediately following WBRT was significantly higher than the fold change in the athymic nude mice 12 h post-WBRT. In the brain, CXCL1 concentrations were significantly increased in WT and nude mice 12 h post-WBRT. When comparing the two strains in relation to their controls, WT mice had a 2.5-fold increase in their CXCL1 brain concentration while nude mice had a 1.5-fold increase. These data suggest the nude mice have a delayed, lower magnitude inflammatory response to WBRT. The relationship between TNF-α and CXCL1 is well documented in the literature [48–51]. In endothelial cells, TNF-α binds to its receptor and initiates the JNK and p38 MAPK signaling pathways to secrete CXCL1 [49]. Additionally, TNF-α increases expression of CXCR2, the receptor of CXCL1, on endothelial cells and enhances adherence of Th17 cells, a proinflammatory subtype of T-helper cells associated with neuroinflammation [50, 51]. Th17 cells then cross the BBB, cause neuronal cell death, and maintain the proinflammatory environment through immune cell recruitment and production of IL-17. Production of IL-17 has been shown to decrease the expression of tight junction proteins in mouse models of multiple sclerosis [48]. We hypothesize this pathway is activated in response to WBRT, leading to BBB disruption in WT but not athymic nude mice.

Our study expands on previous work in the field by highlighting the impact of the immune response to WBRT on BBB dysfunction, however, there are a few limitations to our work. First, our studies were completed using a single dose of radiation at 15.5 Gy. Although this is a similar BED to the clinical dosing of 30 Gy in 10 fractions, the clinical dosing schedule may have differential effects. To determine the extent of altered efflux transporter activity, competitive inhibition experiments are needed. More work is necessary to elucidate the underlying mechanism of BBB disruption following WBRT in immunocompetent mice. Our previous work demonstrates BTB disruption in athymic nude mice post-WBRT, therefore, more research is required to determine the differences in WBRT-mediated BTB and BBB disruption with immunocompetent and immunocompromised mouse modeling.

## Conclusion

The BBB is disrupted following WBRT, but the extent and timing of disruption vary between studies. It is necessary to understand factors which may be contributing to altered BBB permeability following irradiation to develop more efficacious treatment strategies when combining radiation with systemic therapy. Our work demonstrates the impact of the immune response to WBRT on BBB permeability in a time- and size-dependent manner. This is relevant in the preclinical cancer research field due to the more frequent use of immunocompromised mice with human cancer cell lines. Our work suggests this may not be an accurate model of BBB permeability. Furthermore, we identified a window of time post-WBRT where efflux transporter activity is significantly decreased. Numerous anti-cancer therapeutics are substrates for efflux transporters, such as doxorubicin, vinblastine, and taxanes [52, 53]. A transient decrease in efflux transporter activity and an increase in BBB permeability may enhance delivery of these therapeutics across the BBB. We also offer a potential mechanism and avenue for further exploration.

## Supplementary Information

**Additional file 1**. **Figure S1**: Nude mice have significantly decreased efflux transporter function, but no differences in BBB integrity. **A**, **B**: No significant differences in ^14^C-sucrose uptake between mouse strains at baseline (**A**). Nude mice had a significant increase in ^3^H-ivermectin uptake at baseline (**B**) (*p* < 0.05).

**Additional file 2**. **. Figure S2**: No changes in BBB permeability following WBRT in immunocompetent mice 3, 6, or 24 h post-WBRT. **A**–**C** No significant differences in ^14^C-AIB uptake observed 3 (**A**), 6 (**B**), or 24 (**C**) hours following WBRT (15.5 Gy) in wild-type C57Bl/6 mice (*p* < 0.05).

**Additional file 3**. **Figure S3**: No changes in BBB permeability following WBRT in immunocompetent mice 3, 6, or 24 h post-WBRT. **A**–**C** No significant differences in 3 kDa TxRd uptake observed 3 (**A**), 6 (**B**), or 24 (**C**) hours following WBRT (15.5 Gy) in wild-type C57Bl/6 mice. (*p* < 0.05).

**Additional file 4**. **Figure S4**: No changes in proinflammatory cytokine concentrations in serum of immunocompetent mice post-WBRT. **A**–**F** No significant differences in IL-1β (**A**), IL-2 (**B**), IL-5 (**C**), IL-6 (**D**), IL-10 (**E**), or CXCL1 (**F**) in WT mice serum immediately, 6 h, or 12 h following WBRT (*p* < 0.05).

**Additional file 5**. **Figure S5**: No changes in proinflammatory cytokine concentrations in serum of immunocompromised mice post-WBRT. **A**–**C** No significant differences in IL-1β (**A**), IL-2 (**B**), or CXCL1 (**C**) in nude mice serum 12 h following WBRT (*p* < 0.05).

**Additional file 6**. **Figure S6**: No changes in proinflammatory cytokine concentrations in brain of immunocompetent mice post-WBRT. **A**–**E** No significant differences in IL-1β (**A**), IL-4 (**B**), IL-5 (**C**), IL-6 (**D**), or IFN-γ (**E**) in WT mice brains immediately, 6 h, or 12 h following WBRT (*p* < 0.05).

**Additional file 7**. **Figure S7**: No changes in proinflammatory cytokine concentrations in brain of immunocompromised mice post-WBRT. **A**–**I** No significant differences in IL-1β (**A**), IL-2 (**B**), IL-4 (**C**), IL-5 (**D**), IL-6 (**E**), IL-10 (**F**), IL-12p70 (**G**), IFN-γ (**H**), or TNF-α (**I**) in athymic nude mice brains 12 h following WBRT (*p* < 0.05).

## Data Availability

The datasets used and/or analyzed for this study are available upon request from the corresponding author.

## Abbreviations

BBB: Blood–brain barrier
BTB: Blood-tumor barrier
AIB: α-Aminoisobutyric acid
WBRT: Whole-brain radiation therapy
P-gp: P-glycoprotein
BCRP: Breast cancer resistance protein
SRS: Stereotactic radiosurgery
BED: Biological effective dose
TxRd: Texas red
WT: Wild-type
