# Supplementary figures and images for: Effects of whole-brain radiation therapy on the blood–brain barrier in immunocompetent and immunocompromised mouse models
Source: Radiat Oncol. 2023 Feb 3;18:22. doi: 10.1186/s13014-023-02215-6 (PMC9896731; doi:10.1186/s13014-023-02215-6)

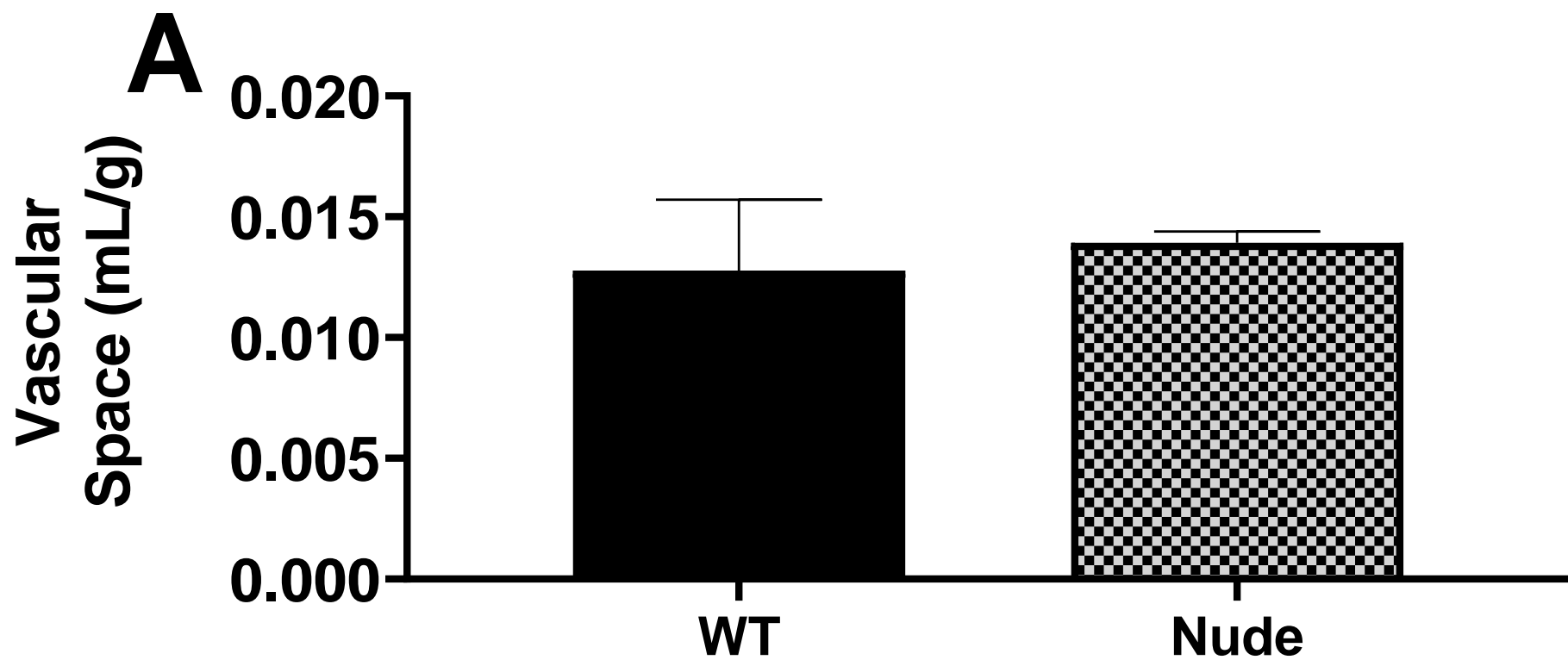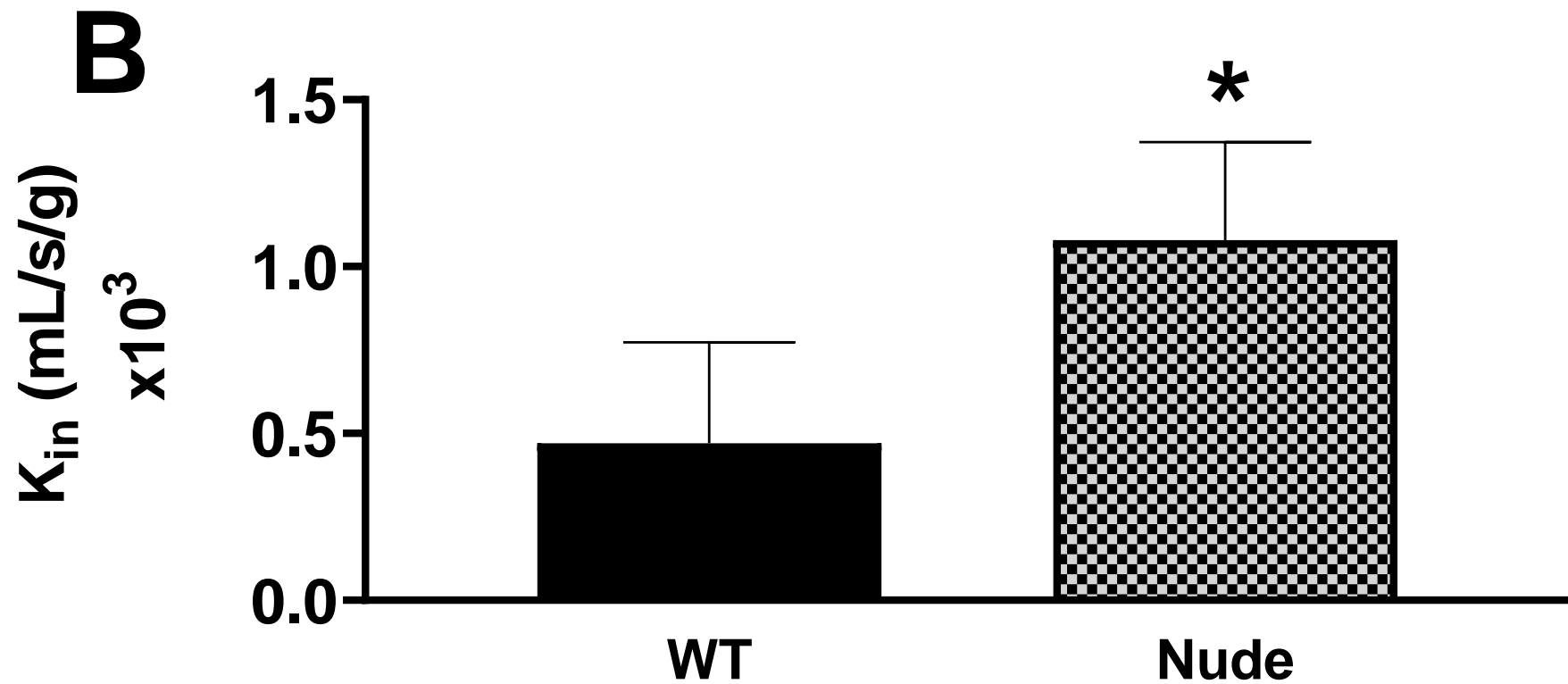

Supplement: Supplementary file 1 — Additional file 1. Figure S1: Nude mice have significantly decreased efflux transporter function, but no differences in BBB integrity. A, B: No significant differences in 14C-sucrose uptake between mouse strains at baseline (A). Nude mice had a significant increase in 3H-ivermectin uptake at baseline (B) (p < 0.05). [file 13014_2023_2215_MOESM1_ESM.pdf]

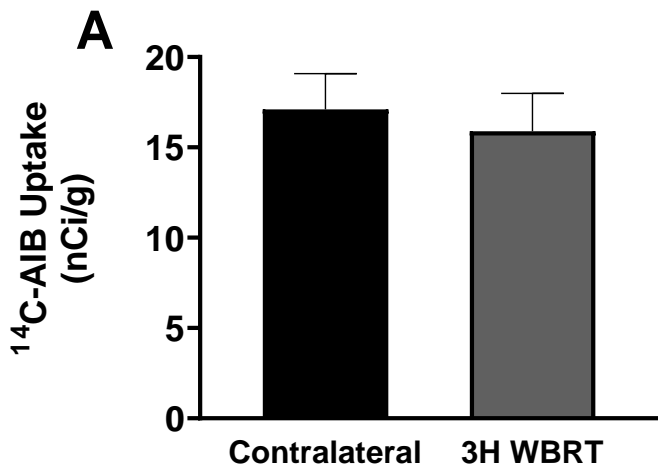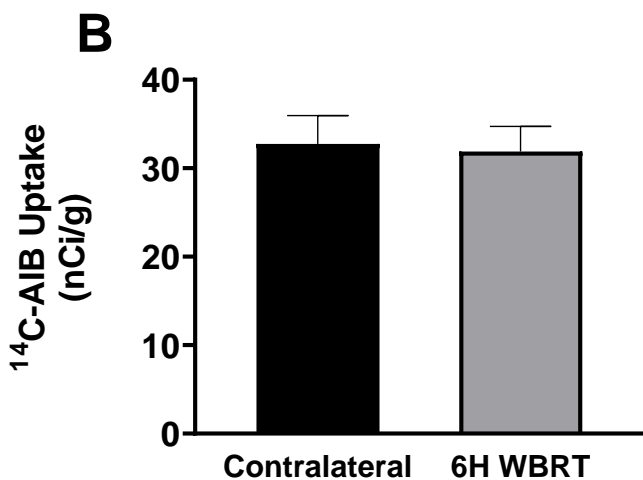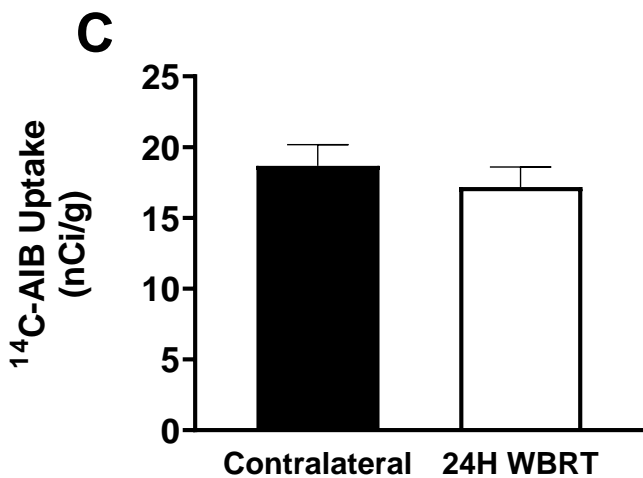

Supplement: Supplementary file 2 — Additional file 2. . Figure S2: No changes in BBB permeability following WBRT in immunocompetent mice 3, 6, or 24 h post-WBRT. A–C No significant differences in 14C-AIB uptake observed 3 (A), 6 (B), or 24 (C) hours following WBRT (15.5 Gy) in wild-type C57Bl/6 mice (p < 0.05). [file 13014_2023_2215_MOESM2_ESM.pdf]

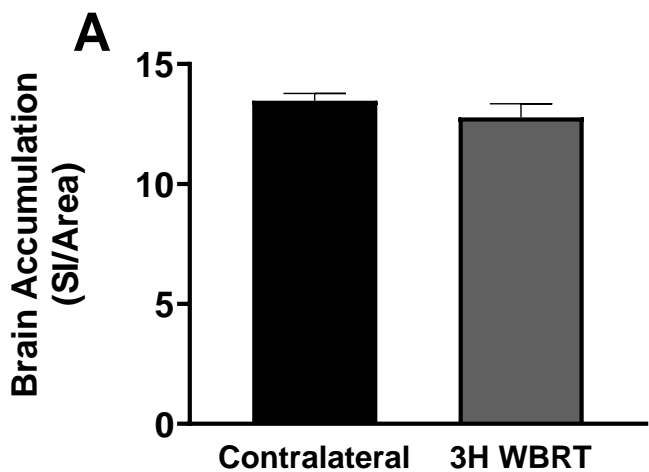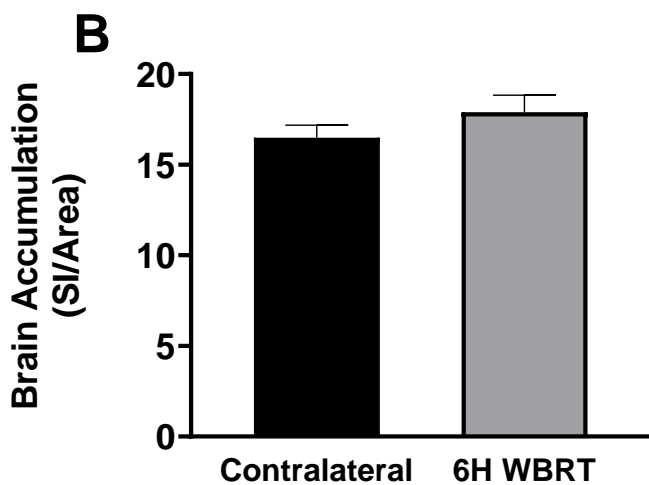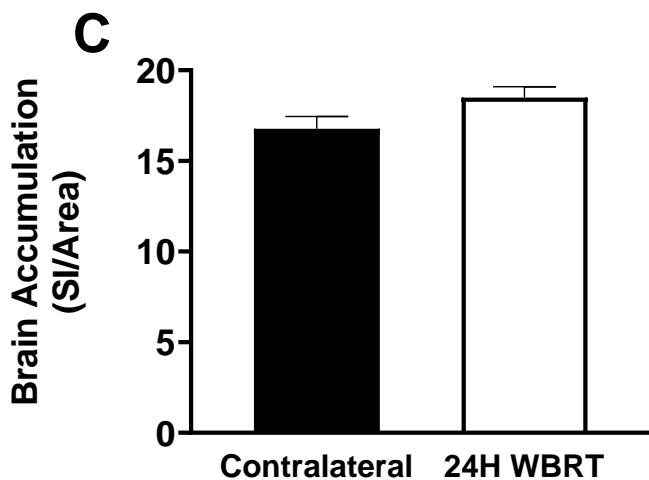

Supplement: Supplementary file 3 — Additional file 3. Figure S3: No changes in BBB permeability following WBRT in immunocompetent mice 3, 6, or 24 h post-WBRT. A–C No significant differences in 3 kDa TxRd uptake observed 3 (A), 6 (B), or 24 (C) hours following WBRT (15.5 Gy) in wild-type C57Bl/6 mice. (p < 0.05). [file 13014_2023_2215_MOESM3_ESM.pdf]

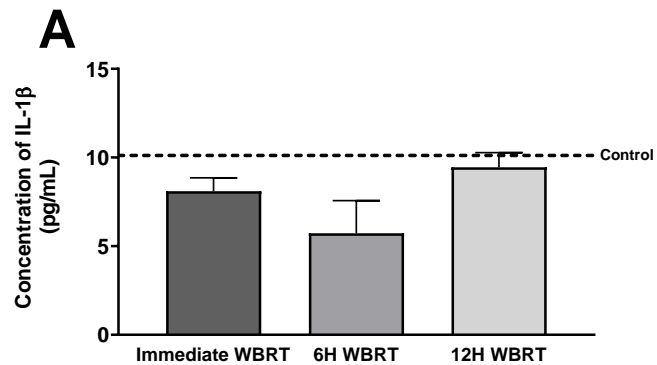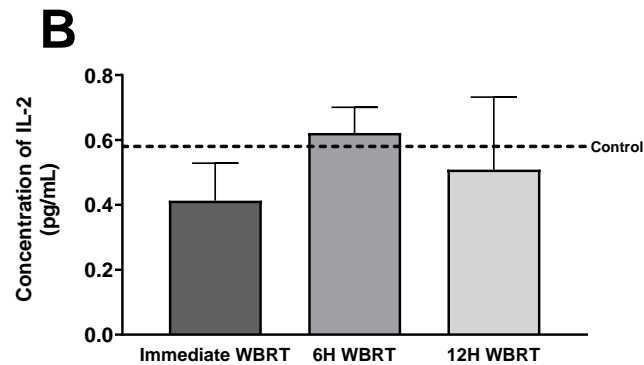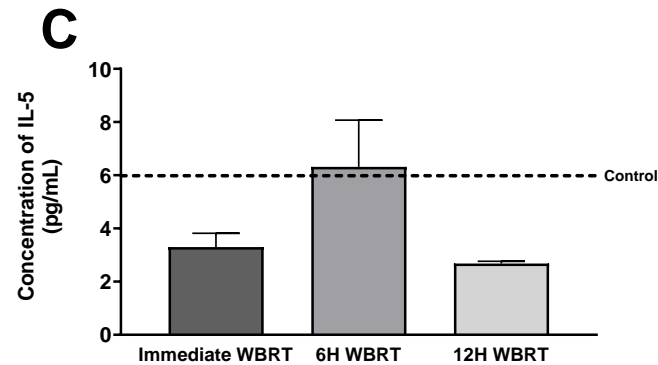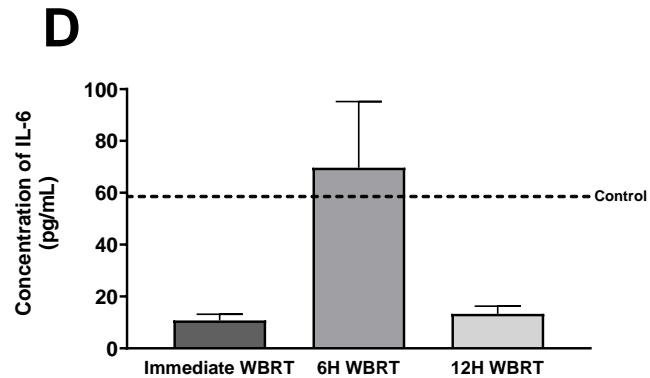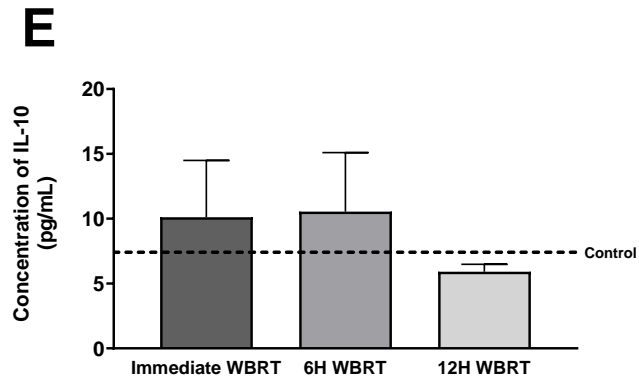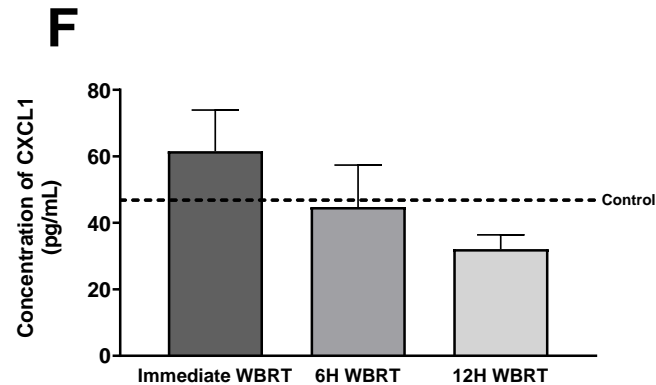

Supplement: Supplementary file 4 — Additional file 4. Figure S4: No changes in proinflammatory cytokine concentrations in serum of immunocompetent mice post-WBRT. A–F No significant differences in IL-1β (A), IL-2 (B), IL-5 (C), IL-6 (D), IL-10 (E), or CXCL1 (F) in WT mice serum immediately, 6 h, or 12 h following WBRT (p < 0.05). [file 13014_2023_2215_MOESM4_ESM.pdf]

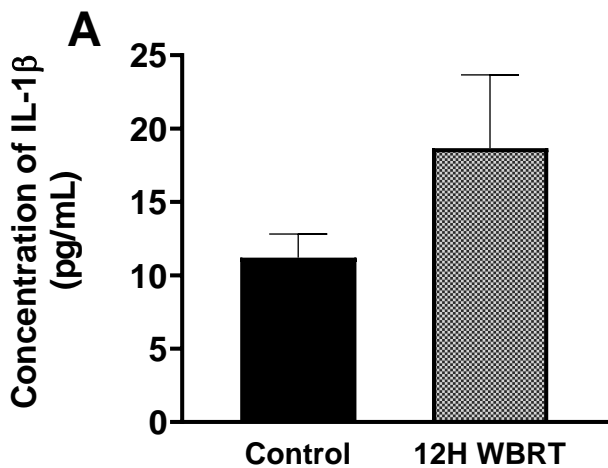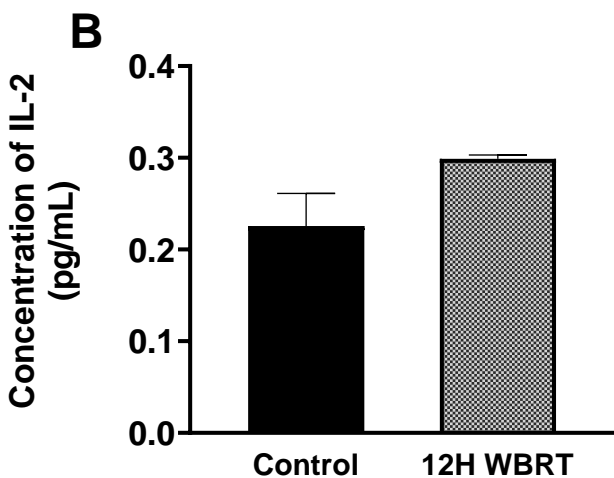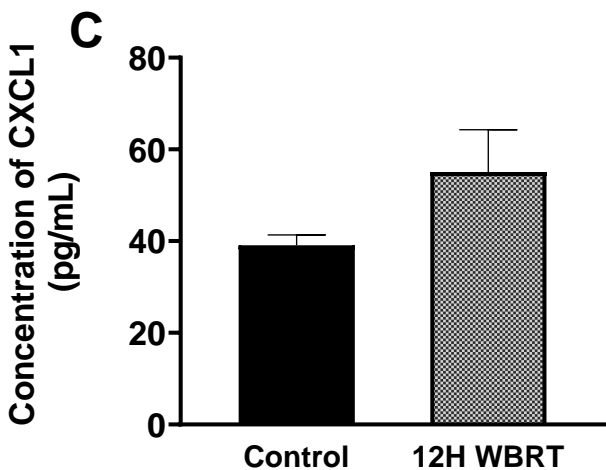

Supplement: Supplementary file 5 — Additional file 5. Figure S5: No changes in proinflammatory cytokine concentrations in serum of immunocompromised mice post-WBRT. A–C No significant differences in IL-1β (A), IL-2 (B), or CXCL1 (C) in nude mice serum 12 h following WBRT (p < 0.05). [file 13014_2023_2215_MOESM5_ESM.pdf]

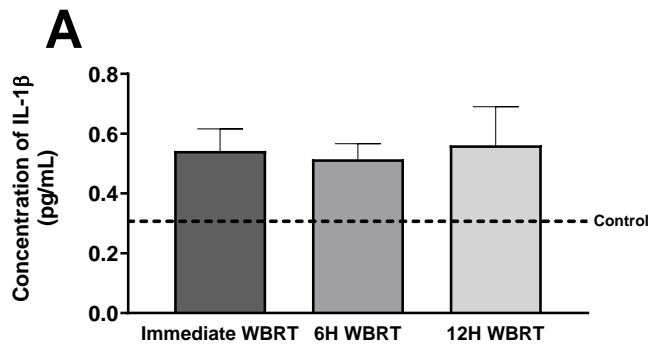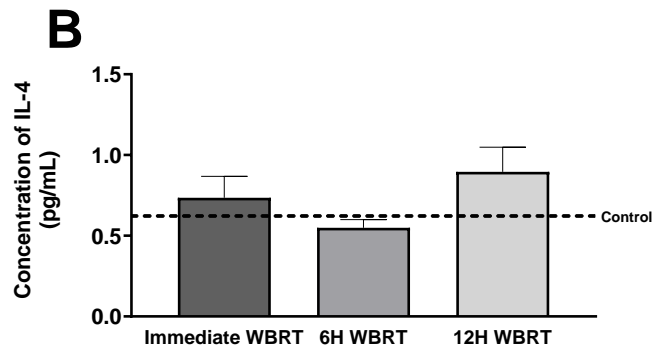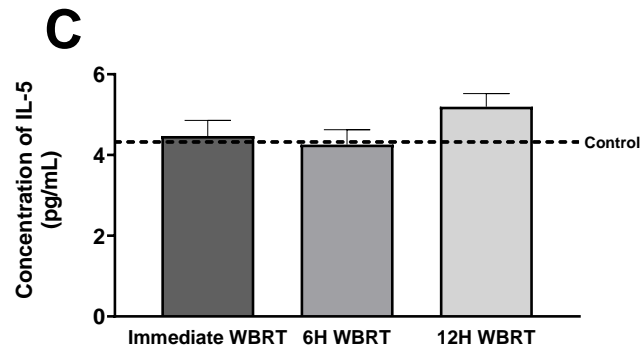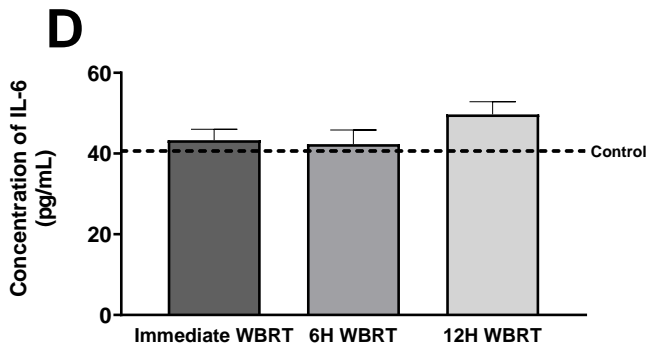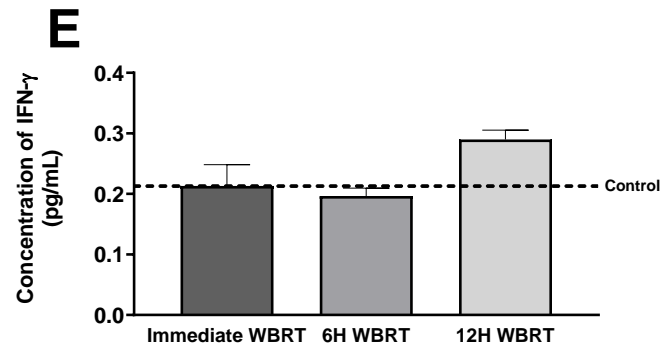

Supplement: Supplementary file 6 — Additional file 6. Figure S6: No changes in proinflammatory cytokine concentrations in brain of immunocompetent mice post-WBRT. A–E No significant differences in IL-1β (A), IL-4 (B), IL-5 (C), IL-6 (D), or IFN-γ (E) in WT mice brains immediately, 6 h, or 12 h following WBRT (p < 0.05). [file 13014_2023_2215_MOESM6_ESM.pdf]

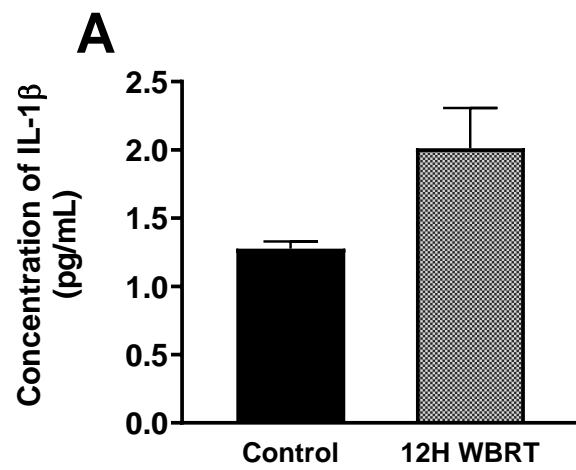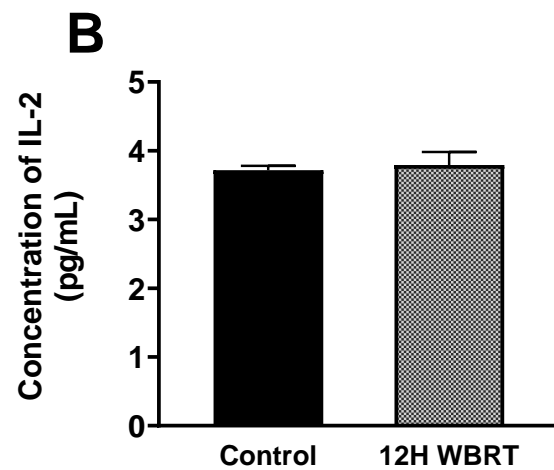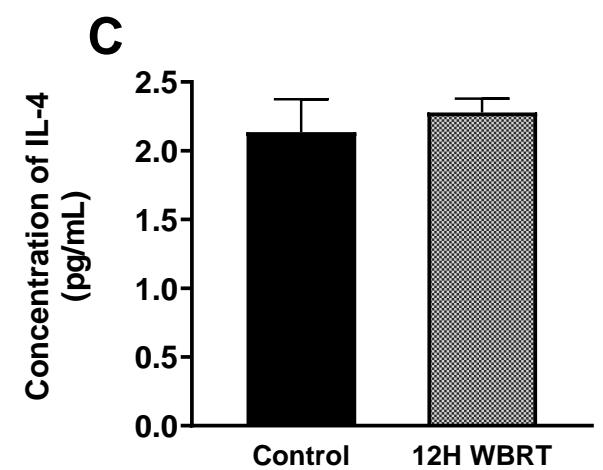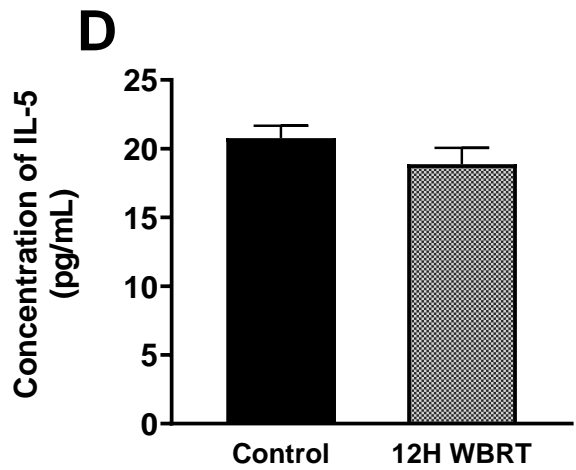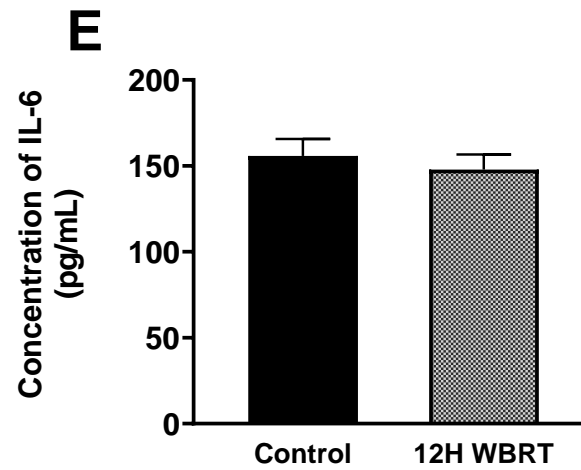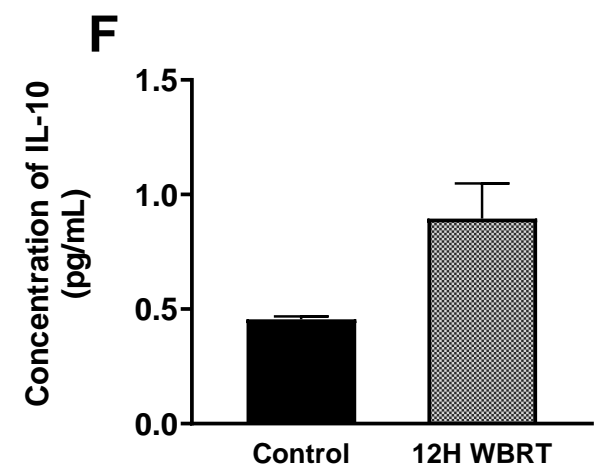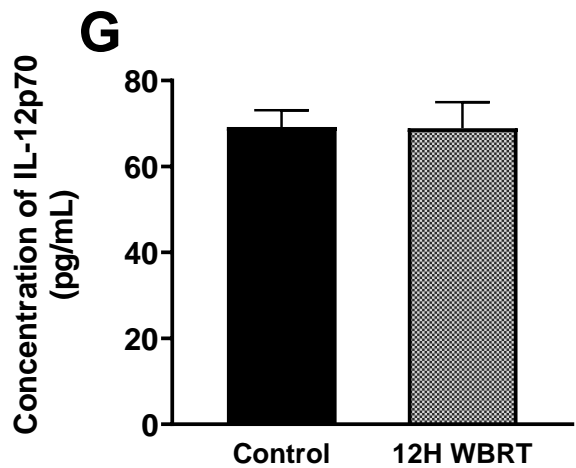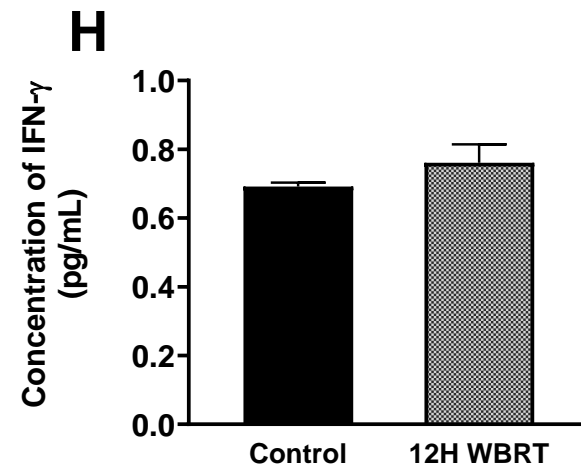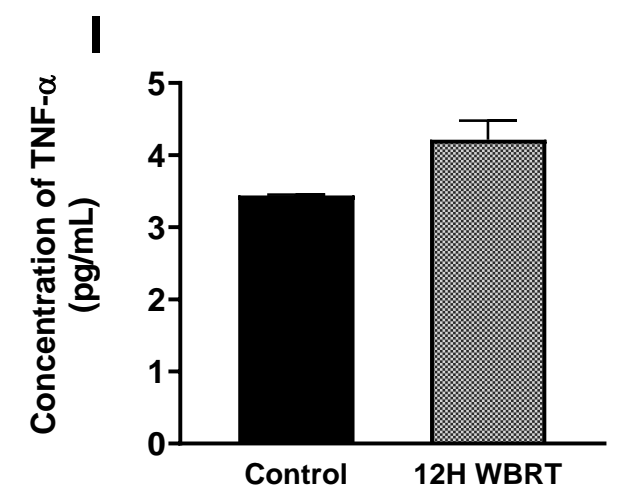

Supplement: Supplementary file 7 — Additional file 7. Figure S7: No changes in proinflammatory cytokine concentrations in brain of immunocompromised mice post-WBRT. A–I No significant differences in IL-1β (A), IL-2 (B), IL-4 (C), IL-5 (D), IL-6 (E), IL-10 (F), IL-12p70 (G), IFN-γ (H), or TNF-α (I) in athymic nude mice brains 12 h following WBRT (p < 0.05). [file 13014_2023_2215_MOESM7_ESM.pdf]
